# Adhesion of Vulcanite to Metallic Bases

**Published:** 1904-12-15

**Authors:** Walter M. Bartlett

**Affiliations:** St. Louis, Mo.


### ADHESION OF VULCANITE TO METALLIC BASES.

BY WALTER M. BARTLETT, D.D.S., ST. LOUIS, MO.

At the beginning of 1840, artificial teeth were mounted almost entirely upon metallic bases by means of soldered backings, or post anchorage. A few years later Dr. Allen introduced continuous gum, which gave to the profession a class of work by which artificial teeth could be mounted upon a platinum base by first soldering the teeth to the base and then fusing around them a porcelain body. With this class of work the restoration of lost contour could be accomplished.

At a later date, vulcanizable rubber was introduced into dentistry, giving to the profession a method of constructing a denture which marked the beginning of a downward trend in the line of dental prosthesis.

Dr. Allen originated a class of work which, when properly constructed, was all that could be desired in the way of art and utility, requiring the best efforts of the skilled mechanic and an artistically-trained eye.

When Dr. Evans made use of vulcanite for the construction of an artificial denture he placed in the field a cheap competitor against the one advocated by Dr. Allen. Before entering upon the work proper to my subject I will touch lightly upon the history of vulcanite.

The exact date of the introduction of vulcanite for dental use is veiled in some doubt, but we know that some time between 1844 and 1855 it first began to be employed. In 1851, Nelson Goodyear's process for making vulcanite was published and turned the attention of manufacturers to the adaptation of this material, which was announced to be a substitute for horn, bone and ivory, susceptible to being colored, and having the plasticity of gutta-percha or caoutchouc, and exempt from the action of heat, cold and acids.

In 1855, Charles Goodyear, Jr., obtained in England a patent for making a dental plate of hard rubber, although it has been claimed that the late Thomas W. Evans, of Paris, France, produced a vulcanite denture in 1845. A lengthy litigation resulted from the claims of the different parties to the priority of the use of vulcanite for dental purposes, which resulted invariably in favor of the Goodyear Company.

To accomplish a result with vulcanite did not require the skill, expenditure and judgment necessary to manipulate the other, and consequently the profession rushed madly to this cheap base, using it entirely, or in combination with metallic bases, for the attachment of teeth.

This method of combining the two has been in use for upward of fifty years, and yet I hardly know of a theory which has been advanced stating why such a combination is possible.

Vulcanite is produced by incorporating a given amount of sulphur with caoutchouc, the proportions varying according to the uses for which the vulcanite is intended after induration. This given amount of sulphur incorporated with caoutchouc to produce the vulcanite used for dental bases no doubt varied in the formulas of different manufacturers. Assuming this to be the case, I endeavored by correspondence to ascertain the amount of sulphur incorporated with the caoutchouc from some of the different manufacturers of vulcanite, but in all cases they were loth to give the information, so in conducting my experiments I was forced to rely upon the formulas given by Dr. E. Wildman and upon my own investigation. During some of my leisure moments I have gone deeply into this subject, carrying out quite a line of experiments to determine the adhesive properties of various metals to vulcanite as utilized for dental purposes.

It was first necessary to supply myself with all those metals necessary for prosthetic work, both practical and technical. Then I had to determine upon some method of obtaining definite practical data in regard to the amount of pressure or weight necessary to bring about a separation of the vulcanite and metal employed. A perfect mold constructed of gun metal was procured, which contained four depressions measuring three-quarters of an inch in diameter by one-quarter of an inch in depth. In the center of the base of the mold a depression was made to receive the eyelet contained upon the disc.

The rubber utilized in these experiments was whalebone. According to a recent analysis made by Dr. Prinz this rubber contained 25 percent sulphur.

The various metals were cut in discs and squares measuring three-quarters of an inch in diameter. With the exception of aluminum and its alloy, tin and its alloy, viz: Watt's metal, these discs were supplied with a soldered eyelet. The rubber was carefully packed into the mold, facing the polished surfaces of these various metal discs, and the so-packed mold was then placed in the vulcanizer and vulcanized at a temperature of 320 degrees for one hour. The object of this experiment was to attach the weight to the combined rubber and metal plate by means of a spring scale, and thus find out the amount of pressure or weight necessary to cause a separation between the two bases to determine the amount of adhesion.

The rubber used in the molds weighed prior to vulcanization 183 grains, after vulcaniaztion 179 grains, indicating a certain loss, which during the process of vulcanization,

| Metals | Weight of Metal | Loss in Metal | Adhesion | Remarks |
|---|---|---|---|---|
| 24 kt. Gold | 22.7 | None | None | Rubber polished, no change on metal. |
| Coin Gold | 20.6 | None | None | Metal and rubber, polished surface. |
| 20 kt. Gold | 20.8 | None | 35 lb | Metal slightly tarnished, rubber polished. |
| 18 kt. Gold | 24.7 | None | 140 lb | Metal tarnished, rubber polished. |
| Pure Silver | 14.3 | 42% | None | Metal partly destroyed, rubber soft and rough surface. |
| Coin Silver | 14.1 | 5% | None | Metal heavily tarnished, rubber soft and rough surface. |
| Platinum | 26.5 | None | None | Metal and rubber highly polished surface. |
| Aluminum, Zeller's | 3.9 | None | None | Metal and rubber polished. |
| Aluminum | 1.6 | None | None | Metal and rubber polished. |
| Magnalium | 21.4 | None | Slight | Metal polished, rubber slightly tarnished and polished. |
| Dental Alloy | 14.7 | None | 5 lb | Metal slightly tarnished, rubber polished. |
| Nickel | 11.7 | None | None | Metal and rubber tarnished. |
| German Silver | 11.7 | None | 7 lb | Metal polished and tarnished, rubber polished and tarnished. |
| Victoria Metal | 12.7 | None | 10 lb | Metal tarnished and polished, rubber tarnished and polished. |
| Iridiumoid | 10.6 | None | None | Metal partially tarnished, rubber partially tarnished. |
| Copper | 9.9 | 33% | None | Metal partially destroyed, rubber soft, heavily tarnished. |
| Brass | 14.4 | None | 10 lb | Metal polished, tarnished surface, rubber partially tarnished and polished. |
| Goldoid | 12.1 | 1.65% | None | Metal tarnished, surface flakes off, rubber tarnished. |
| Weston's Metal | 52.5 | None | None | Metal tarnished, polished, rubber highly polished. |
| Tin | 63.7 | None | None | Metal tarnished and polished, rubber polished. |

being practically a dry distillation, can only be accounted for in this loss of its specific gravity, being simply a liberation of sulphuretted hydrogen resulting from the action of the hydrogen atoms of the hydrocarbon upon the sulphur present in the vulcanite. Quite an important change in regard to the weight of certain metals before and after vulcanization occurred.

From this table two important factors can be deducted:

First: Loss of weight in certain metals.

Second: The amount of adhesion between certain metals and vulcanite.

Practically only two metals, viz: silver and copper, showed, according to the above table, a material loss. Pure silver lost 42 percent, coin silver 5 percent, and copper 33 percent. Goldoid, an alloy of copper and nickel, lost 1.65 percent. The loss of silver during process of vulcanization is a well-known fact. This phenomenon may be simply explained from a chemical point of view, by the fact that sulphur in the form of sulphuretted hydrogen possesses a great affinity for silver. As we have stated above, during vulcanization, dental rubber liberates sulphuretted hydrogen in *statu nascendi,* which in turn attacks the silver metal base, thus rendering it unfit for practical purposes. This same chemical reaction, only in a slightly milder degree, occurs when rubber is vulcanized upon a copper base. The free sulphuretted hydrogen will destroy the copper and form sulphide of copper, which, analogous to the above-formed sulphide of silver, will prevent the rubber from becoming "vulcanized," leaving it in a peculiar soft consistency.

It is rather remarkable to observe that in coin silver, being an alloy of nine parts of silver and one part of copper, this loss amounted to only 5 percent, while each individual metal lost the following percent, viz: Pure silver lost 42 percent, and pure copper lost 33 percent. Dental alloy, consisting of approximately one-third of platinum and two-thirds of silver, and much used as a metallic base for dentures in England and the Continental Europe, showed *no* loss.

The significance of the loss of copper is of less importance to the dental practitioner, as this metal is not utilized for artificial substitutes in connection with vulcanites. However, it is of importance to the teacher of dental prosthesis, as the copper is quite frequently employed in the technic teaching in dental colleges.

Another remarkable fact developed in this connection in regard to the loss of copper and silver under the above treatment. While copper and silver lost quite a large percent when employed separately, that same loss was greatly modified when alloyed together, as in the form of coin silver.

A second and, from a practical standpoint, more important factor can be further deducted from our table. It is the relative amount of adhesion formed between the vulcanite and the metal bases. By looking over our table we find the following data recorded:

18 K. gold plate—140 lb., weight necessary to break adhesion.

German Silver—7 lb., weight necessary to break adhesion.

Victoria Metal—10 lb., weight necessary to break adhesion.

Brass—10 lb., weight necessary to break adhesion.

Dental Alloy—5 lb., weight necessary to break adhesion.

The above are alloys possessing approximately the following composition:

| | | Percent. |
|---|---|---|
| 18 K. Gold Plate | Pure Gold | 75 |
| | Silver | 15 |
| | Copper | 10 |
| German Silver | Copper | 58 |
| | Zinc | 30 |
| | Nickel | 12 |
| Victoria Metal | Copper | 50 |
| | Zinc | 30 |
| | Nickel | 20 |

| | | Percent. |
|---|---|---|
| Dental Alloy | Platinum | 33⅓ |
| | Silver | 66⅔ |
| Brass | Copper | |
| | Zinc | |

If we look over this table very carefully we are forced to conclude that a certain amount of silver, or a certain amount of copper, or an alloy of both in the approximate proportions of two parts of silver to one of copper, must be added to those metal bases which form no union with vulcanite, viz: gold, platinum, nickel, etc., if we wish to bring about an adhesion between the metal base and the rubber. If we keep in mind that neither silver nor copper will unite with rubber, nay, will be partly destroyed in itself and prevent even the rubber from becoming thoroughly hardened during vulcanization, it seems, *a priori*, paradoxical to attribute to these metals a favorable influence upon the union of the two substances. Considering, however, a metal base alloyed with a silver-copper to the extent of about 25 percent, in its union with vulcanite from a chemical standpoint, we may become convinced of the soundness of the following theory which I offer in explanation of this remarkable phenomenon:

During the process of vulcanization of rubber upon metallic bases, other than silver and copper, but alloyed with these two metals in certain proportions, the liberated sulphuretted hydrogen, in its nascent state, acts upon this silver and copper alloy, forming respectively silver and copper sulphides. This chemical removal of the alloy from the metal base will leave the surface of the base in a more or less pitted condition and this fresh rough surface offers a suitable place for the ready adhesion of the vulcanite to the metal. Whether the sulphides of silver or copper in their *statu nascendi* are instrumental in bringing about a closer union, is, in my mind, doubtful. If this were the case, vulcanization upon pure silver and pure copper would not be failures.

From the above experiments we find that there is sufficient adhesion of vulcanite to 20 and 18 K. gold for all practical use were it not for the fact that there is in time a change occurring in the vulcanite which, as it contracts upon itself, becomes detached from the metal base, so to obtain the best results it is necessary to aid adhesion of vulcanite to the metal base by roughening the contact of the base, attaching soldered pins or loops so they can be finally imbedded in the indurated rubber. Great care should be taken in placing the wire finish both labially and lingually to form an underhold for the lodgment of the vulcanite.

In conclusion I wish to extend my thanks to the firms of Arnold Biber and Hans Schmidt, of Germany, for their kindness in so generously sending me materials which I could not well procure in this country and which were indispensable in my line of experiments.—*Review.*

